# Breast Cancer Metabolism and Mitochondrial Activity: The Possibility of Chemoprevention with Metformin

**DOI:** 10.1155/2015/972193

**Published:** 2015-10-28

**Authors:** Massimiliano Cazzaniga, Bernardo Bonanni

**Affiliations:** Division of Cancer Prevention and Genetics, European Institute of Oncology, 20141 Milan, Italy

## Abstract

Metabolic reprogramming refers to the ability of cancer cells to alter their metabolism in order to support the increased energy request due to continuous growth, rapid proliferation, and other characteristics typical of neoplastic cells. It has long been believed that the increase of metabolic request was independent of the mitochondrial action but recently we know that mitochondrial activity together with metabolism plays a pivotal role in the regulation of the energy needed for tumor cell growth and proliferation. For these reasons the mitochondria pathways could be a new target for therapeutic and chemopreventive intervention. Metformin in particular is actually considered a promising agent against mitochondrial activity thanks to its ability to inhibit the mitochondrial complex I.

## 1. Introduction

Although breast cancer is considered a genetic disease in which several mutations and genome dynamic changes are present [1], recent research endeavors are geared to try and understand other mechanisms contributing to the (formation) development and progression of the disease. In this regard, the evidence of the changes affecting cancer cells metabolism has proved to be one of the most promising features and it has influenced several studies on this topic. In spite of this, however, how it works and what this cellular metabolic reprogramming does have long remained unclear [2]. To increase the proliferative activity cancer cell typically needs to modify its metabolic pathways giving rise to a metabolic reprogramming which is generally explained by the metabolic shift from mitochondrial oxidative phosphorylation (OXPHOS) to aerobic glycolysis (Warburg effect) [3, 4]. In particular, while the energy production for metabolic activities in normal cells derives from OXPHOS, an efficient pathway able to produce the adenosine triphosphate (ATP) request, the principal metabolic difference observed in cancer cells is their enhanced avidity for glucose and its consequent strong increase in aerobic glycolysis to fulfill the high-energy demand [5]. In the 1920s, Warburg hypothesized that this shift on glycolysis was the consequence of an altered oxidative metabolism and, in particular, the result of an impairment of mitochondrial activity [6]. This concept has radically changed thanks to the demonstration that mitochondrial activity and OXPHOS efficiency are unchanged also in cancer cells [7, 8] and it means that in every cancer cell, including breast cancer, there is a strong cooperation between the two different pathways in order to produce the energy request. However, in malignant diseases, several pathways concur to shift to aerobic glycolysis involving genetic factors, hypoxia, and tissue microenvironment [9]. The evidence that a part of energy leads to cancer growth still arises by OXPHOS; this means that new or old drug intervention should interfere with the carcinogenetic process and/or the progression of the tumor.

## 2. Normal and Tumor Cells Metabolism

Metabolism is the process whereby biochemicals, oxygen, and nutrients are turned over to generate energy in the form of ATP needed to perform cellular functions or utilized for macromolecular synthesis [10]. Recently, metabolic activities have reemerged as a process able to generate other multiple cellular responses. This is particularly evident in cancer as well as in normal cells function. During their evolution and with the increased availability of oxygen, body cells rely on two different pathways (glycolysis and OXPHOS) to generate ATP and to produce energy [11]. Between these pathways, there are a cooperative relationship and interchangeability producing ATP as a response to different energy request within the cell.

**Figure 1:**
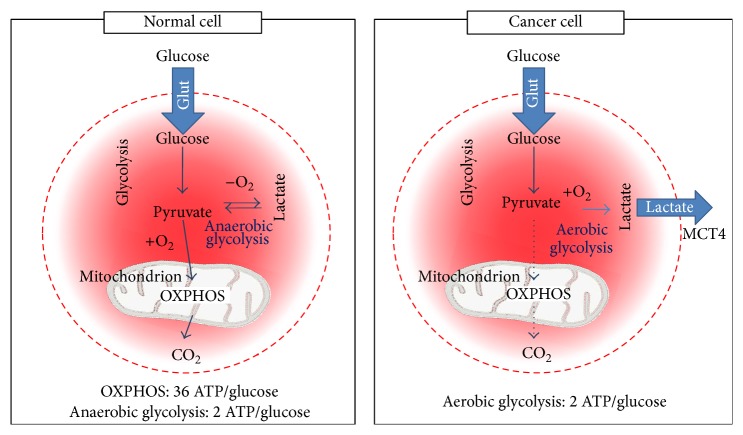
Metabolic differences between normal and cancer cells. Normal cells primarily utilize the OXPHOS process generating 36 ATPs per glucose for its activity. On the contrary, cancer cells convert glucose to lactate (Warburg effect) generating only two ATPs per glucose.

In normal conditions, both pathways contribute to produce energy. Seventy percent (70%) of the request is supplied by OXPHOS, while the glycolysis process ensures the rest of the energy generating 2 ATP molecules by metabolizing the glucose in the cytoplasm. As a result, pyruvate is produced, an important substrate for OXPHOS. In the presence of oxygen, pyruvate enters the mitochondria to be oxidated and to produce 36 ATP molecules [12] (Figure 1). Moreover, other fuels, such as fatty acids, ketone bodies, and amino acids, also support OXPHOS [13]. Therefore, in normal conditions the two different pathways (glycolysis and OXPHOS) are involved in the energy production required to maintain cellular energetic balance.

In addition, this cooperation works under hypoxia where the increased level of glycolysis compensates OXPHOS weakened function [14]. On the other hand, in every cancer cell, including breast cancer, the ratio between the two pathways is overturned and glycolysis becomes the major source of energy, especially in case of hypoxia. For these reasons, this process is called aerobic glycolysis (or Warburg effect) and is considered a worse prognostic factor in cancer settings [15]. Glycolysis is a better way for ATP production in cancer tissues because it is more suitable for cancer cells growth and a higher production of energy may worsen the situation [16]. Although glycolysis yields less ATP than OXPHOS, this action is quicker and more suited for a proliferating tissue as in cancer tissues [17]. Tumor cells are fully dependent on an adequate energy supply in order to support cellular events, such as growth, proliferation, migration, and invasion. For instance, proliferation alone encompasses several anabolic reactions, all of them energetically expensive; this condition requires a deep reprogramming in order to guarantee an energy increase [18]. Moreover, in premalignant diseases, there is a consequent development of hypoxia and acidosis conditions [19] and glycolysis offers cellular growth advantage under a lower pressure of oxygen state. The major regulatory mechanism of aerobic glycolysis in hypoxia involves the hypoxia-inducible factor (HIF-1), which is a master regulator of several genes and glycolytic enzymes markedly different compared to those in unaffected cells. Moreover, it is involved in several biological processes including metabolism, angiogenesis, metastatic ability, resistance to chemotherapy, and a generally increased cancer severity [20, 21]. As a consequence of the enhanced glycolysis, a large amount of lactic acid is produced in cancer cells, generating a toxic environment [22]. This acidosis condition selects for resistant phenotypes that maintain higher invasion and motility properties [23, 24] overincreasing mitochondrial activity [25]. However, the mechanism of metabolic reprogramming is not yet fully understood, although it is now increasingly clear that a number of oncogenes and tumor suppressors contribute to this phenomenon. The PI3K/Akt/mTORC1 signaling axis, for example, is a key regulator of aerobic glycolysis and biosynthesis, driving the surface expression of nutrient transporters and the upregulation of glycolytic enzymes [26]. Although the glucose avidity of cancer cells is widely demonstrated, this pathway is not the only source of energy present in this setting. For instance, another major change in cancer cells involves glutamine metabolism.

**Figure 2:**
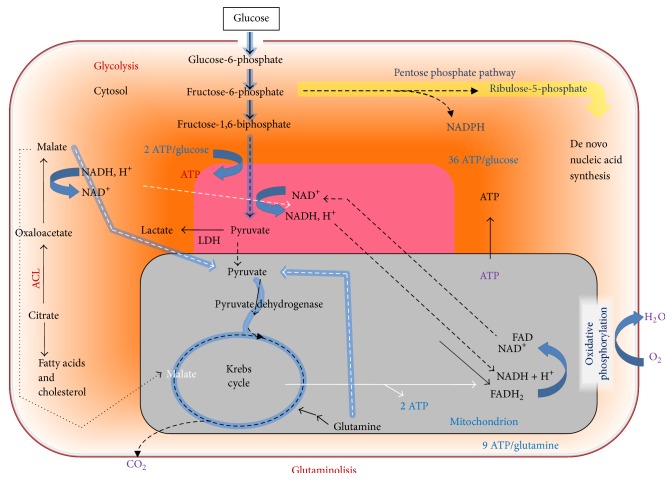
Glutaminolysis pathway and its relationship with other different energy fuel pathways in cancer cells.

Glutamine is a key nutrient for numerous intracellular processes, including oxidative metabolism and ATP generation. Although most mammalian cells are capable of synthesizing glutamine, the demand for this amino acid can become so high during rapid proliferation, as in cancer conditions, that an additional extracellular supply is required [27, 28]. Interestingly, the glutamine pathway is largely dependent on a mitochondrial enzyme (glutaminase). The importance of glutamine for many critical processes in cancer cells and the fact that glutamine metabolism is regulated by both oncogenes and tumor suppressors [29–31] makes this branch of cancer metabolism another attractive target for therapeutic strategies, in particular involving mitochondrial activity, glutamine being a high-energy mitochondrial fuel (Figure 2).

## 3. Mitochondrial Activity in Cancer Cells

As previously mentioned, at the beginning of the century, Dr. Warburg hypothesized that the increased aerobic glycolysis activity in neoplastic cells was the result of a dysfunction of the mitochondrial activity [6]. Although several studies were performed to confirm the weakness of this hypothesis, the considerable effort in this field essentially obtained negative results [32], partially due to the lack of knowledge about mitochondrial biology and behavior in cancer settings.

Thus, OXPHOS upregulation remains a common feature in human cancer, giving the opportunity to utilize mitochondrial activity as a new target for cancer therapy. Recently, a new inhibitor of mitochondrial protein translation seems to be promising in this field [33]. It is clear that cancer cells are addicted to glutamine, a powerful and recognized ingredient for high-energy mitochondrial action [34]. Thus, cancer cells seem to depend on the mitochondrial activity as for the energy required and they need a healthy mitochondrial condition for their reprogrammed metabolism [35]. However, mitochondria are not only the energy generators, but also the factories where many indispensable molecules are synthesized for cellular biosynthesis, growth, and proliferation.

From a biological point of view, mitochondrial activity is fundamental for several biochemical pathways, in particular for bioenergetic and apoptosis-related pathways, and it is clear that its dysfunction may cause a long list of human diseases, including cancer [36, 37]. Moreover, mitochondrial activity is involved in early tumorigenesis and in the acquisition of malignant phenotypes. The fact that several common characteristics of tumor cells are directly or indirectly related to mitochondrial deregulation confirms this assumption.

Several studies performed in this setting have obtained controversial results with the evidence of OXPHOS reduction or upregulation in different cancers that were analyzed. Though, these apparently conflicting data seem to be related to tumor size, presence of hypoxia, and activated oncogenes [38–40]. However, the importance of mitochondria in a dynamic view of tumor energetic characteristics seems undoubtful. Moreover, authoritative studies have recently indicated that modulating mitochondrial respiratory chain can achieve an arrest of cancer cell proliferation, growth, and progression, and, ultimately, it can also achieve anticancer effects.

In comparison with healthy and differentiated cells, cancer cells frequently rewire their mitochondria to switch from a maximal energy production by means of the mitochondrial electron transport chain to a well-adjusted balance among constant energy requirement.

In conclusion, glycolysis and mitochondrial activity seem to create a perfect symbiosis in cancer cells. They cooperate to ensure their survival and if glycolysis is clearly fundamental for cancer metabolism, mitochondrial activity helps cells to adapt to hostile microenvironments. The mitochondrial action gives to cancer cells a useful metabolic flexibility, for instance, allowing high level ATP production. This metabolic complexity is well-established by the conflicting results obtained in several preclinical and clinical studies utilizing mitochondrial inhibitors in therapeutic settings [41–44].

Finally, mitochondria are both the “powerhouse” and the “Achilles' heel” of cancer cells. Hence, the increase in mitochondrial biogenesis is a significant advantage for cancer hence impairing their function and activity, while the lack of their biogenesis may seriously suppress tumorigenesis and cancer growth.

## 4. Targeting Metabolism for Breast Cancer Treatment and Prevention: The Possibility of Metformin

The reprogrammed metabolism supporting cancer cell proliferation and survival leaves the cells vulnerable to therapeutic strategies that disrupt metabolic hallmarks of the transformed state. There is substantial evidence that other conditions (i.e., obesity, hyperglycemia, and hyperinsulinemia) play a fundamental role in cancer development, progression, and prognosis [45], and these pathways are actually considered a target of new therapeutic strategies. Patients with these conditions show an increased cancer risk [46], including breast cancer risk [47]. Indeed, several agents targeting cancer cell metabolism have already been approved or administered in clinical trials [48, 49]. In particular, several recent epidemiological and clinical studies suggest that the antidiabetic drug metformin seems to be able to prevent the onset and the progression of most types of human cancers, breast cancer included [50–52]. Metformin is a drug widely used to treat patients with type II diabetes mellitus, but also in presence of metabolic syndrome and polycystic ovary syndrome and also in diabetes prevention settings [53] but recently many studies have tried to correlate its action with an antitumor effect. These studies, very different from each other, have, for many reasons, obtained controversial but promising results which seems to be appropriate in order to consider metformin a worthy agent of investigation in this field [50–52, 54, 55].

Thus, how metformin acts on cancer cells and how it may diminish tumor growth are not fully understood and the results obtained by works done in order to clarify this particular setting are controversial. There are generally two hypothesized mechanisms by which it may work. An indirect effect of metformin on carcinogenesis is where, in presence of hyperinsulinemia and insulin resistance state, it reduces systemic glucose levels directly acting in the liver, hence aging directly on insulin, a recognized mitogen for cancer cells, and consequently limiting tumor growth and progression [56]. Thus, this drug may work on the cancer tissue with no need to accumulate in the cells. On the contrary the second mechanism works by means of a direct effect of the drug on breast cancer cells.

About the first hypothesized pathway, a recent excellent work [57] that tried to clarify how metformin works* in vivo* explained that it exerts a significant part of its indirect antitumor effects on breast cancer by lowering serum insulin. In this neoadjuvant WOP trial, researchers have shown how a short-term administration of metformin seems to be able to significantly decrease the insulin receptor (IR) levels on breast cancer tissue and this suggests how insulin-dependent effects could be important in the clinical setting. Moreover, other tumorigenesis-related elements (i.e., inflammatory cells, sex hormones, cytokines, adipokines, growth factors, and metabolic intermediates) could also be affected by metformin.

In contrast of these, the second hypothesized mechanism works by means of a direct effect of metformin on carcinogenesis. Several findings support the fact that metformin may act directly on cancer cells. Recently, the precise mode of action has been clarified: it involves AMPK activation by means of an LKB1-dependent mechanism [58]. LKB1 is a tumor suppressor gene with relevance to epithelial neoplasia; in particular, its activity loss is frequently associated with a syndrome, named Peutz-Jeghers, characterized by several gastrointestinal polyps and by a significantly increased risk of various epithelial cancers, including breast cancer [59]. According to several published studies, AMPK activation strongly suppresses cell proliferation in both malignant and nonmalignant cells. A recent excellent WOP trial [60] has shown an upregulation of pAMPK (a phosphorylated AMP-activated protein kinase serving as an energy sensor) and suppression of insulin responses suggesting a cytostatic metformin's mechanism of action. The presence of inactive or inefficient LKB1-AMPK pathways increases the metabolic changes that occur in premalignant cells [61]. In this scenario, many tumors have been shown to negatively regulate the Warburg effect and, in general, the metabolic reprogramming with a negative effect on tumor growth* in vivo* [62].

However, not all the aspects about the relationship between metformin and its anticancer activity have been clarified. For instance, it is still unclear whether AMPK activation is really essential for metformin activity because its ability to inhibit mTORC1 has been demonstrated, also in AMPK-independent pathways [63].

Moreover, LKB1 gene status may be predictive of tumor cell fate upon metformin exposure [64], where* in vivo* altered LKB1 activity may cause neoplastic cell death through their increased sensitization to metformin-induced energy stress [65]. As with LKB1, the role of p53 in cellular metabolic behavior is complex and somehow contradictory [66, 67]. For these reasons, an effective anticancer therapeutic strategy should target the whole tumor complex, including several pathways and characteristics of epithelial cancer cells, cancer stem cells, and the microenvironment, in particular stromal cells.

The latter is a consequence of the oxidative stress metabolites released by the tumor cells and is affected by the so-called “reverse Warburg effect” with a direct supply of lactate and ketones to cancer cells (by aerobic glycolysis) thus increasing their energetic metabolism [68]. In this scenario, it is easy for epithelial cancer cells to behave as parasites and feed these high levels of metabolites in order to guarantee adequate and efficient ATP production via mitochondrial OXPHOS [69]. In this context, the ability of metformin to prevent cancer is likely to stem in its antimitochondrial activity [44] and, in particular, in its ability to hit the cancer stem cells which prefer to use OXPHOS [70, 71].

Recently it has been also proposed that different pathways may help us to clarify the anticancer action of metformin [72], suggesting a direct effect on aromatase activity, while [73] hypothesized an involvement of the Sonic hedgehog (Shh) signaling pathway regularly related in changes in mammary ducts and malignant transformation.

Anyway, these various, controversial but promising results, which seem to be consistent with beneficial anticancer effects of metformin, could be important to identify the key factors involved in sensitivity as well as determining candidate biomarkers in large clinical trials of metformin [74] in order to evaluate the real efficacy of the drug in adjuvant setting and finally could be used to select a cohort of patients with breast cancer who may be responsive to metformin-based therapies.

In particular, several randomized phase I–III clinical trials have been done or are currently ongoing in order to test the efficacy of metformin for breast cancer. Besides the already mentioned NCIC CTG MA.32 trial it is important to report other recent randomized studies recently performed in this field [52, 75–78] and although the data must be taken with chariness, they seem to confirm the metformin's anticancer effect also in clinical setting.

## 5. Action of Metformin on Mitochondrial Activity

These data show that metformin action on cell metabolism is still controversial. We can summarize it into two opposite pathways, depending on the presence of the intact LKB1-AMPK axis. In detail, in presence of intact AMPK, metformin can counteract the Warburg effect of the preneoplastic cells and the presence of this active pathway gives the tumor cells an advantage by protecting them against energetic stress. Conversely, the absence of this axis makes cancer cells selectively more vulnerable to depleted ATP incurred by metformin, as their ability to restore energy balance is impaired.

Thus an alternative pathway directly on cancer cells was recently shown, involving mitochondrial activity. Metformin could target cancer cells directly by cutting the energy supply produced by their mitochondria. So far, there is substantial evidence that the activity of mitochondrial complex I appears to be amplified in breast cancer epithelial cells and its aberrant activity can profoundly enhance the progression and the aggressiveness of the disease [79]. Metformin has been shown to inhibit complex I of the electron transport chain, used by these cells to produce energy, and it acts as a weak “mitochondrial poison” inhibiting oxidative phosphorylation [80]. Its inhibition implies a reduced ATP synthesis, an increased ADP : ATP, and AMP : ATP ratios, hence an indirect AMPK activation. Moreover, the reduction of metformin-induced mitochondrial activity makes stimulated cells take up more glucose; this also results in a therapeutic effect; that is, it lowers blood glucose levels.

**Figure 3:**
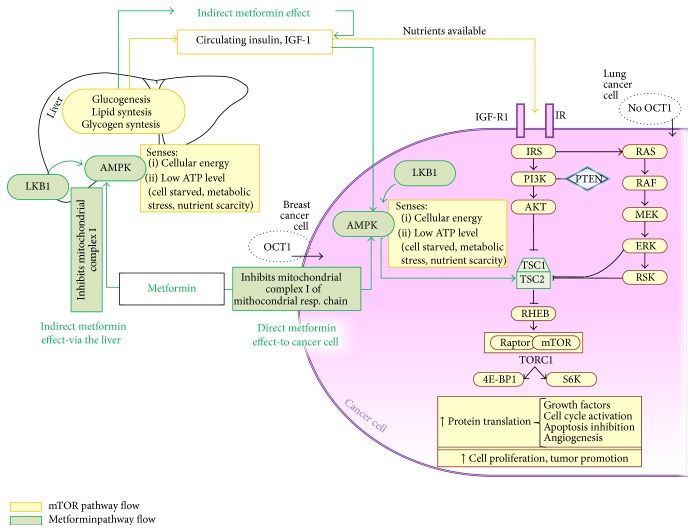
Complete metformin mechanism of action with emphasis on its inhibitory effect on mitochondrial activity.

Researchers have demonstrated that metformin does target complex I in cancer cells and that its effects depend on the amount of glucose available for cells to convert, without involving mitochondria, into energy [81]. In presence of abundant amounts of glucose, metformin slows down the rate of cancer cell division and consequently it slows down tumor growth, while in deprived glucose conditions metformin kills cancer cells. The results in these settings highlight the importance of mitochondrial complex I inhibition of cancer cells as a major mechanism through which metformin reduces tumor burden. Despite this, it does not necessarily preclude any additional organismal effects of metformin, such as the hepatic gluconeogenesis inhibition that might indirectly reduce tumor progression (Figure 3). The levels of metformin within cells are regulated by a balance between expulsion mechanism and mechanisms favoring the drug uptake. The uptake mechanisms are dependent on expression of organic cation transporters (OCT1-OCT2 and OCT3) and mitochondrial membrane potential, while the expression of multidrug and toxin extrusion proteins (MATE 1-2) regulates the opposite effect [82]. In order to inhibit mitochondrial complex I and consequently decrease tumorigenesis, cancer cells need a robust inner mitochondrial membrane potential to allow metformin to reach the mitochondrial matrix. It is therefore possible to hypothesize that metformin efficacy as an anticancer agent depends on the tumor expression of OCTs and a recent work [83] has shown that the selectivity of metformin response may be due to hormonal, metabolic, and associated genetic factors, including some allelic polymorphisms related to OCTs. Moreover, cancer heterogeneity of OCT1, and hence for cancer cell uptake of metformin, could be assessed before considering metformin therapy in the clinical and prevention setting given, for example, the low expression of OCT1 in normal breast tissues. The potential effects of polymorphisms of OCT1, OCT2, and OCT3 on resistance to metformin and interactions between proton pump inhibitors and metformin via OCT1, OCT2, and OCT3 [84] will clearly need to be considered when metformin translates into routine clinical practice. Metformin also inhibits the pathways regulating hypoxia-inducible factors (HIFs) [85] which are, as we have already seen, part of a system that helps cells survive in low-oxygen conditions, as it happens in tumor cells. This could mean that metformin may fight cancer more effectively when associated with a treatment able to reduce availability of both oxygen (e.g., angiogenic inhibitors) and glucose (e.g., PI3K inhibitors) within cancer cells.

## 6. Conclusions

The metabolic activities in normal cells mainly rely on mitochondrial oxidative phosphorylation (OXPHOS) for energy generation by ATP production. Conversely, in cancer cells they predominantly rely on aerobic glycolysis rather than OXPHOS. For a long time it was believed that this switch was due to an impairment of mitochondrial activity. However, an intact mitochondrial activity in cancer cells has been recently shown and various forms of metabolism utilized by neoplastic cells have also been observed. These metabolic pathways are obviously attractive targets for possible therapeutic interventions and currently under investigation. In this scenario, the use of metformin as a mitochondrial inhibitor should be considered as an optimal compound for breast cancer prevention and treatment and to limit drug resistance which is the major cause of conventional treatment failure in cancer patients.

As such, understanding the specific role of mitochondrial dysfunction in cancer pathogenesis may be an interesting and fundamental target for new anticancer therapies and preventing or limiting the onset of conventional drug resistance in cancer patients.
